# The *Lymantria dispar* IPLB-Ld652Y Cell Line Transcriptome Comprises Diverse Virus-Associated Transcripts

**DOI:** 10.3390/v3112339

**Published:** 2011-11-21

**Authors:** Michael E. Sparks, Dawn E. Gundersen-Rindal

**Affiliations:** USDA-ARS Invasive Insect Biocontrol and Behavior Laboratory, 10300 Baltimore Ave., Bldg 011A, Rm 214, BARC-West, Beltsville, MD 20705, USA; E-Mail: Michael.Sparks@ars.usda.gov

**Keywords:** RNA-Seq, EST, transcriptomics, IPLB-Ld652Y, Lepidoptera

## Abstract

The enhanced viral susceptibility of the gypsy moth (*Lymantria dispar*)-derived IPLB-Ld652Y cell line has made it a popular *in vitro* system for studying virus-related phenomena in the Lepidoptera. Using both single-pass EST sequencing and 454-based pyrosequencing, a transcriptomic library of 14,368 putatively unique transcripts (PUTs) was produced comprising 8,476,050 high-quality, informative bases. The gene content of the IPLB-Ld652Y transcriptome was broadly assessed via comparison with the NCBI non-redundant protein database, and more detailed functional annotation was inferred by comparison to the Swiss-Prot subset of UniProtKB. In addition to *L. dispar* cellular transcripts, a diverse array of both RNA and DNA virus-associated transcripts was identified within the dataset, suggestive of a high level of viral expression and activity in IPLB-Ld652Y cells. These sequence resources will provide a sound basis for developing testable experimental hypotheses by insect virologists, and suggest a number of avenues for potential research.

## 1. Introduction

The *Lymantria dispar* (gypsy moth) cell line, IPLB-Ld652Y, was originally derived from pupal ovary tissue by Goodwin *et al*. over three decades ago [1]. This cell line distinctly exhibits enhanced viral susceptibility, which has rendered it a popular *in vitro* system for studying virus effects in the Lepidoptera, particularly those mediated by nucleopolyhedroviruses [2–7] and baculovirus-like viruses [8]. Despite its utility towards facilitating molecular virology research in insects, this cell line has not yet been characterized from a transcriptomics perspective. No mRNA sequence libraries for Ld652Y have been made publicly available to date, nor do transcriptome or genome resources exist for the cell line’s host, *L. dispar*. As of August 2011, fewer than 40 distinct *L. dispar* mRNA sequences could be retrieved from the GenBank nucleotide database, and no transcript reads had yet been deposited to either the dbEST [9] or SRA [10] repositories. In the present communication, the gypsy moth transcriptome is profiled via the IPLB-Ld652Y cell line using both a survey of single-pass EST and 454-based reads derived from uninfected IPLB-Ld652Y cells (hereafter referred to as Ld652Y). This is the fourth established lepidopteran cell line cDNA library, as Landais *et al.* [11] and Deng *et al.* [12] generated cDNA libraries for *Spodoptera frugiperda* (fall armyworm) cell lines SF-9 and SF-21, respectively, and Okano *et al.* [13] profiled ESTs from the *Bombyx mori* cell line BmN. In particular, the Ld652Y-based cDNA library is the first to be generated using next generation sequencing technologies. Given the relevance and widespread utilization of Ld652Y cells for elucidating mechanisms of viral infectivity, these sequence resources will likely be of significant value to the insect virology community.

## 2. Results and Discussion

A collection of 14,368 Putatively Unique Transcripts (PUTs) was compared to the NCBI NR protein database using Blastx (see the Experimental Section below). 6,524 (≈45.4%) of these exhibited a best hit in NR satisfying moderately stringent parsing criteria. (5,069 of these NR hits were unique: Multiple PUTs may correspond to a particular NR gene, because their constituent transcript reads lack sufficient overlap to allow merging during assembly.) Although a number of PUTs corresponded to housekeeping genes such as ribosomal proteins (384) and proteasome subunits (73), numerous immune- and apoptosis-related genes that could potentially explain the response of gypsy moth larvae to baculovirus infection at the molecular genetic level were identified [5,6]. For example, we observed relatively high expression of a cyclophilin A gene, which has been implicated in conferring increased susceptibility to viral infection by HIV-1 in an *in vitro* context [14,15]. In addition, an annexin IX signal transduction factor was observed, which has been implicated in programmed cell death in the anterior silk gland of *Bombyx mori* [16]; apoptotic genes are known to play a significant role in baculoviral infection of lepidopteran cell lines [17,18].

Although beyond the scope of this report, this transcript collection will be useful for further exploration of virus-related phenomena in Ld652Y. For example, gypsy moth Cathepsin B, Actin A3 and Gloverin precursor transcripts, all of which are likely involved in silkworm anti-microbial immune response to infection by the nucleopolyhedrovirus BmNPV [19], are present in the dataset. Similarly, 29 aminoacyl tRNA synthetase transcripts were identified; these might facilitate research confirming the role of defective or depleted tRNA in global arrest of protein translation in AcNPV-infected Ld652Y cells, as has been previously hypothesized [3]. Heat shock cognate protein 70 (Hsc70)—a gene posited to play a key role in baculovirus infection of the *Spodoptera frugiperda* cell lines SF-9 [20] and SF-21 [21] and upregulated in infected *Heliothis virescens* [22]—was expressed at relatively high levels.

Many best hits among the NR Blastx results mapped to virus-related entries. Table 1 depicts the 20 viruses having the greatest number of sequences in the NR database exhibiting similarity to one or more Ld652Y putative unique transcripts, or “PUTs”. In particular, a substantial number of hits corresponded to gene sequences from retroviruses, including human immunodeficiency virus (HIV-1; 444 distinct entries), simian immunodeficiency virus (21 entries) and equine infectious anemia virus (37 entries). It is presently unclear whether these Ld652Y transcripts were expressed from retroviruses or from other retroelements sharing the typical suite of gag-pol-env genes (e.g., retrotransposons or Ty3/Gypsy LTR retroelements)—though the closest hits mapped to the Retroviridae family, suggesting a *bona fide* viral origin of these messages. These results are entirely consistent with the observation of endogenous retroviruses (errantiviruses) in both the SF-9 and Hi-5 lepidopteran cell lines derived from *Spodoptera frugiperda* and *Trichoplusia ni*, respectively [23]. DNA virus sequences were also encountered, some of which had significant similarity to the *Poxviridae*: Cowpox virus, Vaccinia virus and Variola virus, among others. A relatively large number of PUTs had hits to numerous genes from two very large-genomed viruses, the *Acanthamoeba polyphaga* mimivirus [24] and *Cafeteria roenbergensis* virus [25].

A number of nucleopolyhedrovirus-related sequences were detected, suggesting that the cell line may support one or more strains of baculovirus. Interestingly, in addition to baculovirus expressed sequences related to large ds DNA insect viruses, polydnaviruses (including both ichnoviruses and bracoviruses) and nudiviruses were detected. These viruses became associated with the progenitor of modern holometabolous insects over 300 million years ago [26]. The Ld652Y cells used in the present analyses were never exposed to any known insect virus repositories, which supports the hypothesis that the gypsy moth genome hosts laterally-transferred insect virus element(s). In fact, the p94 gene, which is shared between bracoviruses and baculoviruses [27], was detected in a preliminary assembly of scaffold genome sequence data obtained for *Lymantria dispar* itself—this result will be further described in a forthcoming report by the authors.

**Figure 1.**
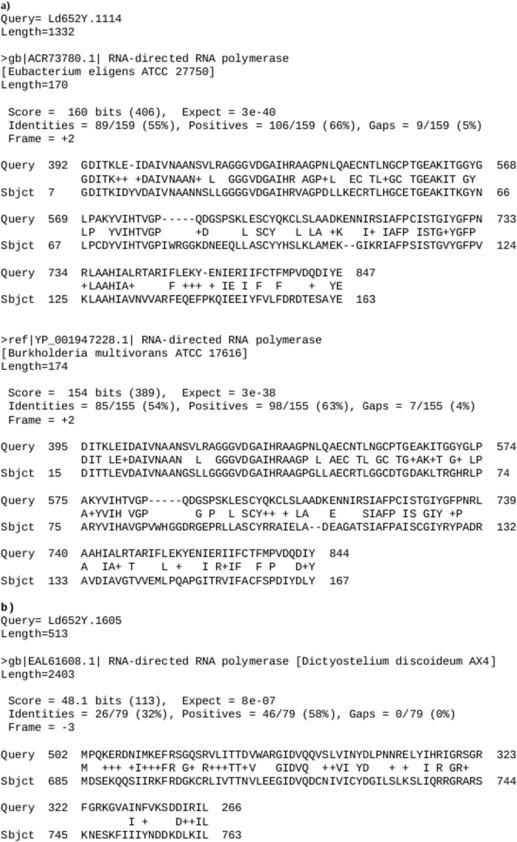
(**a**) Comparison of PUT 1114 to RdRps identified in *Eubacterium eligens* and *Burkholderia multivorans*. (**b**) Comparison of PUT 1605 with an RdRp identified in *Dictyostelium discoideum*.

Katsuma *et al.* noted the expression of RNA viruses (macula-like latent viruses) in the *Bombyx mori* BmN cell line, which were not encoded by the host genome [28]. If persistent RNA viruses are also present and expressed in Ld652Y cells, this might help explain the increased susceptibility of these cells to viral infection. The PUT collection was probed with the RNA replicase and coat protein sequences enumerated in Table 1 of Katsuma *et al.* using Blastx, though no conclusive homologies were observed (data not shown). However, a comparison against a set of RNA-dependent RNA polymerase (RdRp) sequences produced a number of alignments worthy of further consideration (see Figure 1)—These included alignments of PUT 1114 to RdRps identified in *Eubacterium eligens* and *Burkholderia multivorans*, and of PUT 1605 to an RdRp from *Dictyostelium discoideum*.

Separate from RdRp sequences *per se*, a number of other RNA virus-related genes were present in the data, including coat and capsid proteins and polyproteins. These results suggest the latent or sub-lethal presence of one or more RNA viruses in the Ld652Y cell line, and that certain of the viral detection methods described in the article by Liu *et al.* in this issue [29] could be used not only for positive confirmation, but also for reconstruction of their RNA viral genomes. Of note, however, is that the Ld652Y transcript library utilized in the present study was primarily designed to enable characterization of mRNA sequences, whereas methods geared expressly for virus discovery and viral genome assembly (e.g., Wu *et al.* [30] and Mi *et al.* [31]) utilize libraries composed by small RNAs [32]. Transcripts apparently encoding proteins associated with RNA interference pathways, including Dicer, Aubergine and Argonaute, were identified among the Ld652Y PUTs, which suggests that construction and analysis of a small RNA library from Ld652Y will likely generate novel viral discoveries.

To gain insight into the repertoire of expressed transcripts in common between Ld652Y and other lepidopteran cell lines, the Ld652Y PUT collection was compared with the 2,367 SF-21 ESTs reported in Deng *et al.* [12] and 2,711 ESTs derived from the *Bombyx mori* BmN cell line by Okano *et al.* [13]. Using tBlastx with an E-value threshold of 10^−15^, only 1,595 (67.4%) of SF-21 ESTs exhibited one or more significant matches with an Ld652Y PUT, of which 569 of these PUTs were unique. That so few SF-21 EST sequences would have been covered by the Ld652Y PUT collection, which is vastly larger in terms of sequence volume, was unexpected. The balance of unmatched SF-21 ESTs (772 sequences) was compared to NR using Blastx, of which only 217 (28.1%) exhibited one or more significant hits with E-values not more than 10^−15^. Similarly, only 1,121 BmN transcripts (41.4%) were covered by the Ld652Y PUTs; of the 1,590 remaining, unmatched transcripts, only 1,016 (63.9%) exhibited a match to NR per the indicated parsing criteria. These results suggest a high degree of transcript novelty in these Lepidopteran cell line transcriptomes.

**Figure 2.**
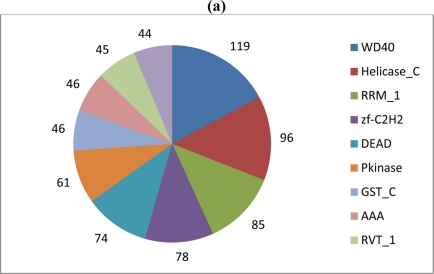

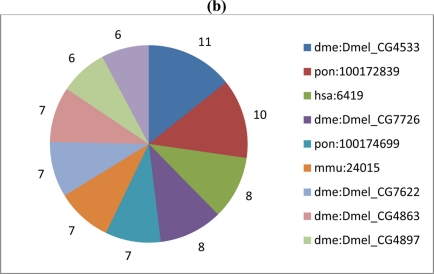
(**a**) Absolute frequencies of the 10 most abundant Pfam protein families encountered in the Ld652Y cell line PUT collection. Counts denote the number of distinct PUTs that encoded the eponymous domain. More complete descriptions of Pfam identifiers are available from the Pfam resource [33]. (**b**) Absolute frequencies of the 10 most abundant KEGG reaction terms encountered by pathway analysis. More complete descriptions of KEGG terms are available from the Kyoto Encyclopedia of Genes and Genomes [34].

The Ld652Y PUT collection was also compared to the Swiss-Prot subset of UniProtKB, which provides a manually annotated and highly reliable source of functional annotation data. 5,243 PUTs (≈36.5%) exhibited a hit satisfying the parsing criteria described in the Experimental Section below, and associated Pfam, KEGG and GO terms were retrieved. 2,107 unique Pfam families were encountered, and a pie chart depicting the 10 most frequently encountered families is shown in Figure 2, panel A. Many of these were for such ordinary families as RNA recognition motifs and glutathione s-transferases. Interestingly, reverse transcriptase proteins (RVT_1) are among the most abundant Pfam families in the dataset, which supports the notion of active expression of, for example, retroviruses/errantiviruses.

3,347 unique KEGG identifiers were retrieved, the 10 most abundant of which are shown in Figure 2, panel B. Perhaps unsurprisingly, the majority of KEGG terms identified corresponded to genes for pathways characterized in *Drosophila*. Finally, GO terms contained in the biological process domain of the Gene Ontology were compiled, with the 25 most abundant terms being listed in Table 2. Ninety-two PUTs were labeled by the ontology as being apoptosis-related, some of which are likely involved in the response of gypsy moth larvae to baculovirus infection.

## 3. Experimental Section

### 3.1. EST Sequence Processing

A normalized IPLB-Ld652Y cell line cDNA library was constructed by Marligen Biosciences (Ijamsville, MD, USA) by directional cloning of size-fractionated double-stranded cDNA into the Express1 vector and transformation into DH10B *E.coli* cells. From a subset of 3,000 isolated cDNA clones, 1,622 EST reads, comprising 1,560,600 bases, were produced using an ABI 3100 sequencing instrument. Base calling was performed using the Phred program [35], and Lucy [36] was used to clip away any remaining contaminant vector sequences, polyA tails and terminal segments of low-quality data. 20-base windows had to exhibit an average Phred score of 17 or better (*i.e.*, not more than a 2.5% error rate) to be retained for analysis, and resultant regions of sufficiently high quality had to span at least 100 continuous nucleotides in length for the read to be retained. The remaining 1,178 trimmed EST sequences were then screened for known Dipteran repetitive elements using RepeatMasker [37] coupled with the most recent version of the RepeatMasker library (dated June 24, 2009 and available from RepBase [38]). Twenty unique sequences harboring such elements were purged from the dataset, and NCBI DUST [39] was used to flag low-complexity sequence. The remaining 1,158 reads were deposited in the dbEST division of GenBank and assigned accession numbers of JK670018-JK671175.

### 3.2. 454 Sequence Processing

Total RNA was harvested from uninfected IPLB-Ld652Y cells, depleted of rRNA, and reverse transcribed using the Evrogen MINT-Universal cDNA synthesis kit. The resulting non-normalized MINT cDNA library was then sequenced using a 454 GS-Junior pyrosequencing system (Roche 454 Life Sciences, Branford, CT, USA) located at the Beltsville Agricultural Research Center (Beltsville, MD, USA). 82,099 raw sequence reads at 100bp or greater in length were deposited into the NCBI Sequence Read Archive under accession number SRA047598.

Reads were trimmed to remove MINT-related adapter sequences [40] on the basis of Blastn alignments [41]. Seven hundred and eighteen reads flagged as repetitive elements by RepeatMasker were eliminated and low-complexity sequences were marked using NCBI DUST. 81,452 reads were retained for assembly, containing 36,521,172 bases of presumably usable information.

### 3.3. Derivation of Putative Unique Transcripts (PUTs)

A hybrid assembly of the 1,158 cleaned EST and 81,452 454-based transcript data was performed using the overlap-layout-consensus algorithm implemented in the cap3 assembly software [42]. This program gave superior results on the dataset from among a panel of assemblers tested (data not shown). 1,202 sequences placed in the singlets pool by cap3 that exhibited a contiguous span of five or more 'N's were eliminated; no such sequences were placed in the contigs pool. The resulting assembly contained 5,924 contigs (comprising 4,836,170 bases) and 8,444 singlets (3,639,880 bases). Contigs and singlets were pooled to define a set of 14,368 putatively unique transcripts (PUTs) containing a grand total of 8,476,050 informative bases.

### 3.4. Gene Content Survey and Functional Analysis

In order to obtain a more defined picture of the gene content captured in the transcriptome data, PUTs were compared with the NCBI non-redundant protein database, NR, using Blastx. Only the top-scoring NR protein encountered in the results was considered, which must have exhibited an E-value not more than 10^−5^. Furthermore, PUTs were aligned to Swiss-Prot, the manually annotated subset of UniProtKB [43], using Blastx. The best hit for each PUT, which also required an expectation value of 10^−5^ or less, was identified and associated with a PUT. Functional annotation data for these best hits—provided by Pfam, GO and KEGG terms—was retrieved and transferred to the PUTs.

## 4. Conclusions

In this paper, the contributions of transcriptome sequence resources inherent to the gypsy moth cell line IPLB-Ld652Y, and germane to the investigation of virus-associated phenomena using this system, were revealed. This is the first such study performed on any cell line derived from *Lymantria dispar*, and these tools are expected to also facilitate discoveries in the host organism itself. The degree to which gene expression observed in these cells reflects that exhibited in an *in vivo* context remains unclear, and is a topic of future consideration. Numerous virus-associated transcripts were identified, indicative of a wide range of viral activity. Among the numerous viral-related transcripts detected were those that mapped to NR protein sequences derived from retrovirus (errantivirus), poxvirus and baculovirus samples. Functional annotation of the Ld652Y transcriptome not only suggested the presence of viral activity, but also helped identify a variety of avenues for future research aimed at elucidating the features of these cells underpinning their virus-susceptible nature. The assembly data and their relevant annotations are available from the authors by request.

## Figures and Tables

**Table 1.** The 20 viruses having the greatest number of NR protein sequences exhibiting hits with IPLB-Ld652Y putative unique transcripts (PUTs).

**Subject Count**	**PUT Count**	**Virus**
444	31	Human immunodeficiency virus 1
111	2	Porcine reproductive and respiratory syndrome virus
89	25	Cowpox virus
63	5	Human herpesvirus 5
60	23	Vaccinia virus
57	54	Acanthamoeba polyphaga mimivirus
37	3	Equine infectious anemia virus
36	1	Beak and feather disease virus
35	13	Variola virus
31	2	Cassava brown streak virus
27	2	Human herpesvirus 1
25	30	Amsacta moorei entomopoxvirus 'L'
24	30	Cafeteria roenbergensis virus BV-PW1
21	9	Simian immunodeficiency virus
20	28	Autographa californica nucleopolyhedrovirus
17	17	Monkeypox virus
17	33	Melanoplus sanguinipes entomopoxvirus
17	18	Fowlpox virus
16	6	Hyposoter fugitivus ichnovirus
15	8	Human herpesvirus 8

Subject count denotes the tally of unique protein sequences from the given virus that registered a hit to one or more PUTs, and PUT count represents the number of PUTs which had hits to the listed virus.

**Table 2.** Absolute frequencies of the 25 most abundant Gene Ontology (GO) terms from the Biological Process domain encountered in the IPLB-Ld652Y cell line PUT collection.

**Count**	**GO Id.**	**Description**
399	GO:0006412	Translation
340	GO:0006351	transcription, DNA-dependent
287	GO:0006355	regulation of transcription, DNA-dependent
140	GO:0006397	mRNA processing
128	GO:0015031	protein transport
128	GO:0006457	protein folding
124	GO:0006810	Transport
110	GO:0008380	RNA splicing
106	GO:0007275	multicellular organismal development
105	GO:0006281	DNA repair
103	GO:0006508	Proteolysis
95	GO:0051301	cell division
95	GO:0007067	Mitosis
95	GO:0006364	rRNA processing
92	GO:0006915	Apoptosis
90	GO:0022900	electron transport chain
64	GO:0000022	mitotic spindle elongation
63	GO:0055085	transmembrane transport
63	GO:0006260	DNA replication
56	GO:0007049	cell cycle
55	GO:0030154	cell differentiation
51	GO:0006886	intracellular protein transport
49	GO:0045454	cell redox homeostasis
48	GO:0007264	small GTPase mediated signal transduction
48	GO:0006511	ubiquitin-dependent protein catabolic process
